# Supplementary figures and images for: Cross-frequency coupling between slow harmonics via the real brainstem oscillators: An in vivo animal study
Source: PLoS One. 2023 Aug 7;18(8):e0289657. doi: 10.1371/journal.pone.0289657 (PMC10406189; doi:10.1371/journal.pone.0289657)

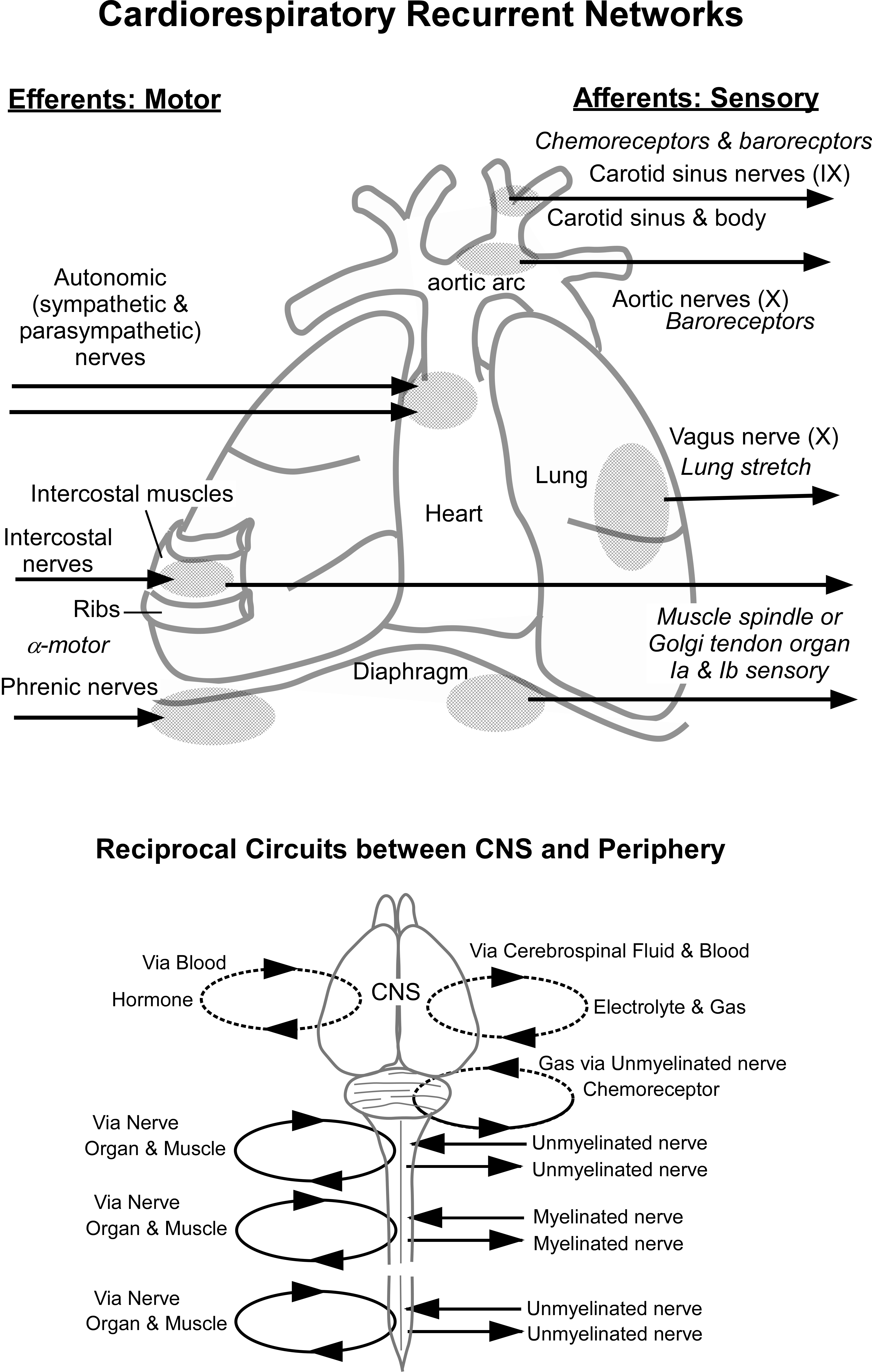

Supplement: S1 Fig — The peripheral networks connected to the CNS involve various organs and muscles via both myelinated and unmyelinated nerves, blood-borne gases, electrolytes, and hormones. Voluntary muscles of respiration involve myelinated efferent and afferent nerves, while involuntary autonomic system both myelinated (preganglionic efferent) and unmyelinated (afferent and postganglionic efferent) peripheral nerves. Note the recurrent networks as oscillators for self-checking and accommodation. Dotted lines of ellipsoids show non-nerve transmission via gasses, electrodes and hormones. Abbreviations: A1-10, C1-3, catecholaminergic cell groups (A: dopaminergic or noradrenergic, C: adrenergic); B1-9, serotonergic cell groups; ACe, amygdaloid central nucleus; AP, area postrema; BNST, bed nucleus of the stria terminalis; Ch, Ch5-6, cholinergic cell groups; DLF, dorsal longitudinal fascicle; H, histaminergic cell group; HPV, hypothalamic paraventricular nucleus; ME, median eminence; MFB, medial forebrain bundle; NTS, nucleus of tractus solitarius; OVLT, organum vasculosum laminae terminalis; PAG, periaqueductal gray; PB, parabrachial nucleus; SFO, subfornical organ. (TIF) [file pone.0289657.s001.tif]

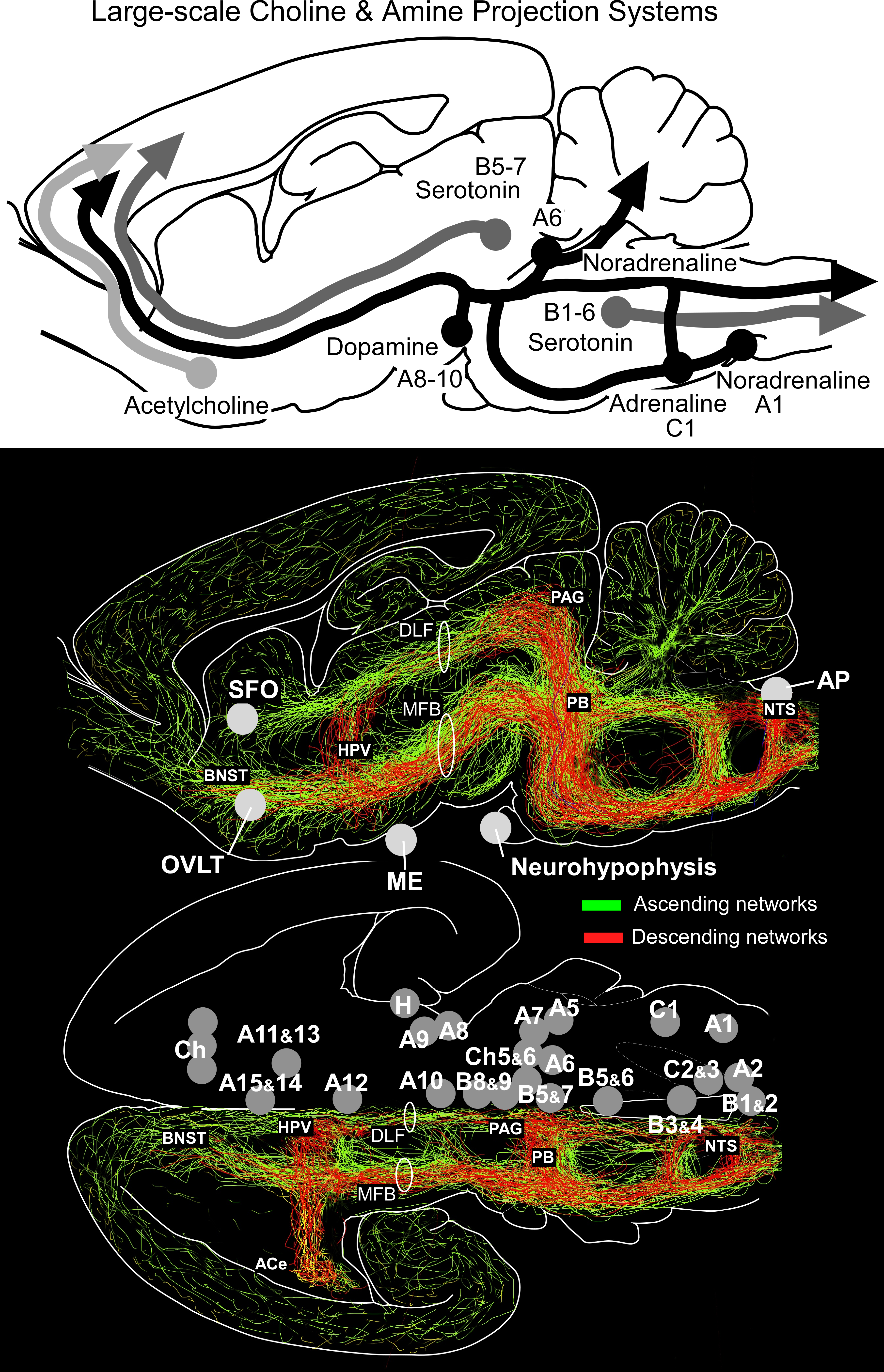

Supplement: S2 Fig — Sagittal and horizontal schematic brain drawings are shown with ascending (green) and descending (red) networks involving the NTS. Reciprocal networks extending from the telencephalon to the spinal cord involve cholinergic (Ch) and aminergic (A, B, C, H: noradrenergic, adrenergic, dopaminergic, serotonergic and histaminergic) neuronal clusters and circumventricular organs (SFO, OVLT, ME, Neurohypophysis, AP), in addition to key nuclei such as PB, PAG, HPV, ACe, BNST via DLF and MFB. These structures may contribute to the maintenance of whole-body homeostasis via nervous and humoral bioregulation. Abbreviations: A1-10, C1-3, catecholaminergic cell groups (A: dopaminergic or noradrenergic, C: adrenergic); B1-9, serotonergic cell groups; ACe, amygdaloid central nucleus; AP, area postrema; BNST, bed nucleus of the stria terminalis; Ch, Ch5-6, cholinergic cell groups; DLF, dorsal longitudinal fascicle; H, histaminergic cell group; HPV, hypothalamic paraventricular nucleus; ME, median eminence; MFB, medial forebrain bundle; NTS, nucleus of tractus solitarius; OVLT, organum vasculosum laminae terminalis; PAG, periaqueductal gray; PB, parabrachial nucleus; SFO, subfornical organ. (TIF) [file pone.0289657.s002.tif]

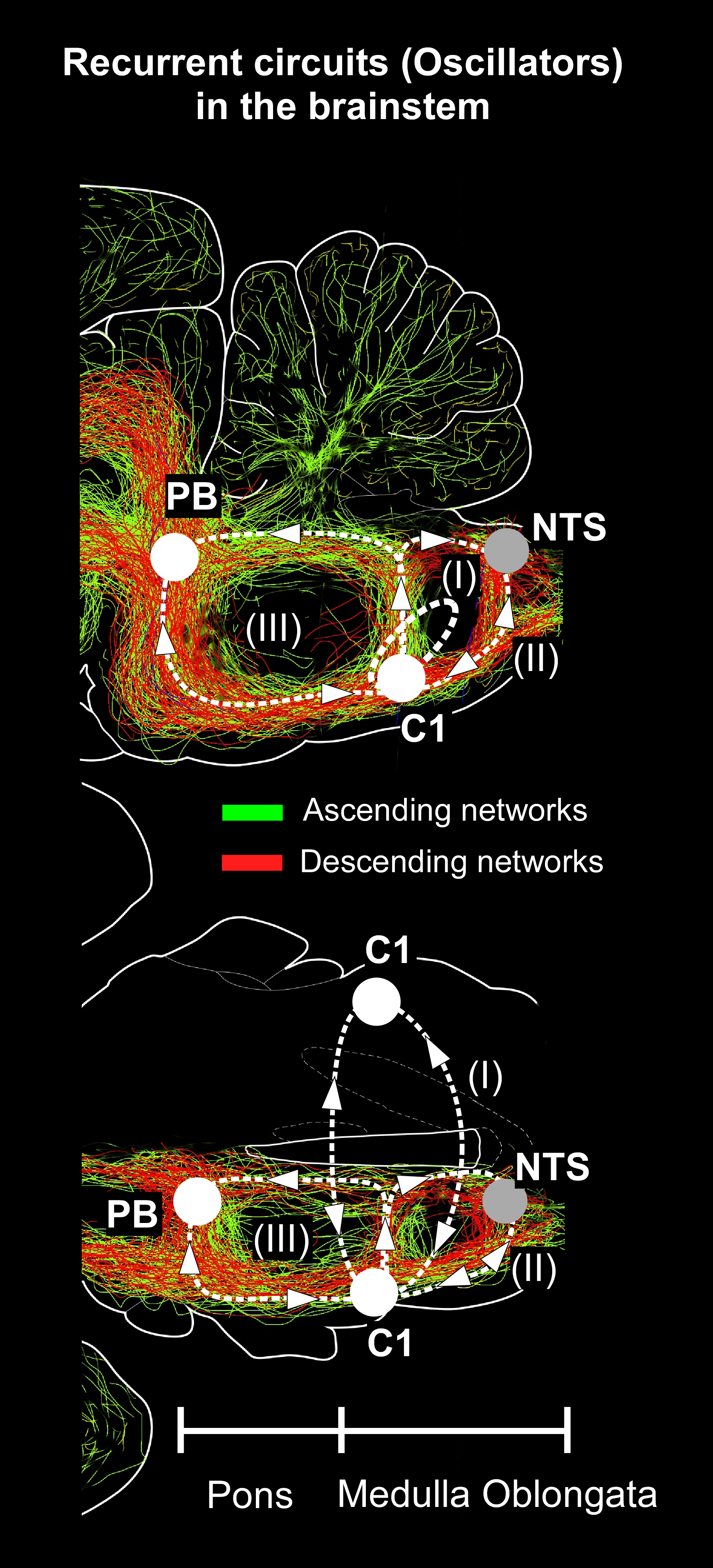

Supplement: S3 Fig — Sagittal and horizontal schematic drawings of the brainstem (pons and medulla oblongata) that contains presumed core oscillators generating delta and theta rhythms are presented. For details of the network configuration in the brainstem, especially the pons and medulla oblongata, see reference [22]. Abbreviations: A1-10, C1-3, catecholaminergic cell groups (A: dopaminergic or noradrenergic, C: adrenergic); B1-9, serotonergic cell groups; ACe, amygdaloid central nucleus; AP, area postrema; BNST, bed nucleus of the stria terminalis; Ch, Ch5-6, cholinergic cell groups; DLF, dorsal longitudinal fascicle; H, histaminergic cell group; HPV, hypothalamic paraventricular nucleus; ME, median eminence; MFB, medial forebrain bundle; NTS, nucleus of tractus solitarius; OVLT, organum vasculosum laminae terminalis; PAG, periaqueductal gray; PB, parabrachial nucleus; SFO, subfornical organ. (TIF) [file pone.0289657.s003.tif]
