# Supplementary material for: Cross-frequency coupling between slow harmonics via the real brainstem oscillators: An in vivo animal study
Source: PLoS One. 2023 Aug 7;18(8):e0289657. doi: 10.1371/journal.pone.0289657 (PMC10406189; doi:10.1371/journal.pone.0289657)
Supplement: Attachment — Submitted filename: Response_to_Reviewer.docx [file pone.0289657.s004.docx]

29 May, 2023

Editorial Office

*PLOS ONE Journal*

Dear Editorial Office:

I wish to submit a revised version of an Original Research Article (PONE-D-23-04711) for publication in *PLOS ONE*, titled “Cross-Frequency Coupling between Slow Harmonics via the Real Brainstem Oscillators: An *in vivo* Animal Study.”

I appreciate the Reviewers’ careful and encouraging suggestions for revision of this article.

In that case, the following revisions or rebuttals (in red in below) were made as our response to comments from the Reviewers.

**Reviewer #1**: The introduction is too brief and relies heavily on the researcher’s own work. Additional references should be included to support the lack of previous research in the area. These could be reviews or articles that mention this gap in the literature.

This is a very important suggestion. Surprisingly, many physiologists of respiration have seemed to believe still that respiratory rhythmic activity is originated in specific neuronal groups in the brainstem as a pace-maker, not neuronal networks, while cardiac one is in the heart, in contrast to my claim that both rhythms of respiration and heart could be generated in the brainstem in a cooperative manner. Few of them have referred to any relationship with cardiac rhythm. Autonomic nervous system physiologists, on the other hand, have suggested that the cardiac rhythm may be associated with the brainstem neurons, however, few have mentioned any relationship with respiratory rhythm. I suspect that clinical cardiologists must recognize the interrelation of cardiorespiratory rhythm but could not yet describe this rhythm relation analytically. Therefore, as far as my knowledge adequate literature concerning cardiorespiratory cooperative activity in terms of rhythmic correlation could not be found for bridging the gap pointed out by this Reviewer. However, most recent references (5&6) concerning respiration networks were added newly with a sentence of “Furthermore, it has been generally stressed that cardiorespiratory rhythms be generated based on pacemaker-like activity originating from particular brainstem neurons, rather than network activity.” in the Introduction of the revised manuscript. (L.49-52)

According to a suggestion of “Please, revise introduction to emphasize the lack of previous research in the area” raised in the mail letter, sentences of “Furthermore, it has been generally stressed that cardiorespiratory rhythms be generated based on pacemaker-like activity originating from particular brainstem neurons, rather than network activity. However, the body-brain interrelationship of rhythmicity….” were incorporated with additional two references (5&6) in the revised manuscript. (LL.49-52)

The sample size (number of rats) needs to be stated. Determining the statistical validity of the findings is difficult without this sample size stated in the materials and methods.

According to the suggestions, number of rats were added in the revised version. (LL.98-99)

The reference section needs revision due to inconsistent formatting. First, there are variations in the listing of the journal’s full name versus abbreviations (ex. 2 vs 4). Second, there are variations in the capitalization of the article name (ex. 4 vs 5). Third, the doi is not always listed even if it is available (ex. 25 vs. 26). The article should be edited to ensure consistency in reference formatting.

The reference list was generated using EndNote with PubMed. However, as pointed out by the Reviewer (#1), the format of listing seems to be not consistent. I have reflected the consistency of the bibliography list notation in the revised version as far as I have noticed, as pointed out by this Reviewer. (LL.685-800)

Several pieces of the paper could be included in the figure legends and vice versa (ex. lines 213-220).

According to the suggestion, that part was changed. (LL.556-564)

**Reviewer #2**: In this paper, the authors investigated brainstem rhythms of the rat and their interactions. One of the rhythms seems to govern respiratory function (delta range) and another rhythm drives heart beats (theta range). It was demonstrated that these rhythms were synchronized in many cases of the brainstem activity by creation of temporary neural circuits. The author found a synchronization between central rhythmic and peripheral cardiorespiratory activities.

In general, the paper is well-written, the results seem solid, and the experimental data supports the conclusions of the paper. However, some sentences in the paper are not clearly written. Basically, writing style must be improved. However, I have only minor points that need to be corrected.

Minor points (lines are numbered as a sequence from the beginning of the paper)

1.Abstract. Too long Abstract. First part, before "In this article …", includes sentences that belong to Introduction.

According to the suggestion, a long sentence in the Abstract, “In this article, by focusing on the physiology and anatomy of a certain rodent brainstem region, the nucleus of the tractus solitarius, we demonstrated the characteristics of harmonic brain waves and their interactions, with a probable anatomical configuration of the responsible oscillator circuits.”, was moved to in the Introduction. (LL.82-85).

2. Line 127 and other epoch durations. I would suggest to provide the number of points of the epochs with the durations of 10, 20, and 100 s that were analyzed. Those are important for the evaluation of the errors in Fast Fourier Transforms.

According to the suggestion, a phrase of “with 1000 points” was added. (L.131)

3. Line 167. What is the meaning of vertical bars in Panels Ba-Be of Fig. 2?

A sentence of “Vertical bars in B indicate relative powers with respect to a common arbitrary unit.” was added in the Figure legends. (L.526)

4. Line 182. 100 sec epoch is not longer than the previously mentioned 1000 sec epoch. Please correct wording. Please also introduce PZT, NTS, and LP in Fig. 3 in the first sentence, such as “Figure 3 shows … and so on”. I think, the reader must observe what is shown in the figure, which is quite complicated.

“A 1000 sec epoch” means a total recording duration of this experiment. From this long epoch recording, epochs of 10-20 sec or 100 sec were selected for FFT analyses.

According to the suggestion, a sentence of “Figure 3 shows two examples of shorter epoch (~10 sec) recordings for coherence analyses between peripheral cardiorespiratory activities recorded by PZT and central neuronal MUAs in the NTS recorded by glass electrodes.” was added. (LL.187-189)

5. Line 188. I assume Figs. A1 and B1 stand for Fig. 3A1 and 3B1?

6. Line 190. There are no panels A2, A3, A4, B2, B3, and B4 in Fig. 2. I assume those are for Fig. 3?

Yes. The relevant figure numbers were carefully checked and all rewritten. (LL.195-209)

7. Line 192. I think "patterns in quality" must be "qualitative patterns"

According to the suggestion, the phrase was changed. (L.199)

8. Line 194. What arrows mean in panels A3 and A4 in Fig. 3?

Yes. The relevant figure numbers were carefully checked and all rewritten. (LL.195-209)

9. Line 213. Standard deviation for Fig. 4Bb seems incorrect. It is of the order of 1.

As the Reviewer pointed out, the digit in the values of the NTS potentials (mV) were wrongly described by one digit. The values were rewritten in the manuscript. The values in the Fig 4B seem to be correct. (LL.220-221)

10. Line 354. Here and further throughout the text: Fig. S1.

According to the suggestion, the words were changed throughout the text. :S# fig. 🡪 Fig. S#. S# and S## figs. 🡪 Figs. S# and S##.

11. Lines 393-395. Bad sentence. Please write more clear sentence or two.

The original sentence of “Brainstem neural activity was confirmed to reflect the fluctuational balance between two distinct oscillators (somatic and visceral: respiratory and autonomic <cardiac>: 0–4 Hz delta and 10 Hz theta).” was rewritten to “The brainstem neural activity could represent a fluctuational cooperation generated by two types of distinct oscillator activities (somatic vs. visceral: respiratory vs. autonomic <cardiac>: 0–4 Hz delta vs. 4–10 Hz theta rhythms).”. (LL.401-403)

12. Line 400. The author can use either FFT or spell it out, but not both. FFT has been defined above.

According to the suggestion, “the fast Fourier transformation (FFT)” was changed to “the FFT”. (L.408)

13. Lines 558-560. I am not clear about the meaning of the last sentence: It looks like coherence disappear, but panels do not show that signals disappear.

The original sentence of “After an apparent cessation of the peripheral and central oscillations, robust signals of delta range almost disappear with theta range signals as seen in (f) – (g).” was changed to “With minor peripheral and central activities of a noise level, robust signals of delta and theta range and their CFC coherence almost disappear as shown in (f) – (g).”. (LL.575-577)

14. Line 613. I would remove "two".

According to the suggestion, the word “two” was removed. (L.630)

15. Line 618. (d) and (q) must be removed.

(d) and (q) should read (δ) and (θ). (L.634-635)

==

I sent the revised version of the original manuscript with “Revised Manuscript with Track Changes” in which changes were shown in red.

Thank you for your consideration. I look forward to hearing from you.

Sincerely,

Yoshinori Kawai, PhD

Adati Institute for Brain Study (AIBS),

Kawaguchi Saitama JAPAN, 333-0811

E-mail: ibs.stm.kwgt@gmail.com
