# Supplementary material for: Cross-frequency coupling between slow harmonics via the real brainstem oscillators: An in vivo animal study
Source: PLoS One. 2023 Aug 7;18(8):e0289657. doi: 10.1371/journal.pone.0289657 (PMC10406189; doi:10.1371/journal.pone.0289657)
Supplement: Attachment — Submitted filename: Response_to_Reviewer.docx [file pone.0289657.s005.docx]

18 June, 2023

Editorial Office

*PLOS ONE Journal*

Dear Editorial Office:

I wish to submit a revised version of an Original Research Article (ONE-D-23-04711R1) for publication in *PLOS ONE*, titled “Cross-Frequency Coupling between Slow Harmonics via the Real Brainstem Oscillators: An *in vivo* Animal Study.”

I appreciate the Reviewers’ careful and encouraging suggestions for revision of this article.

In that case, the following revisions or rebuttals (in red in below) were made as our response to comments from the Reviewer.

Please, make sure that the abstract summarizes the most important results and their significance and does not exceed 300 words.

**1.** Abstract. I suggested to shorten the first part of the Abstract, before the words "In this article …". However, the author moved to the Introduction the sentence started from "In this article …". As the results, Abstract does not describe what is done in the paper. Please shorten the first part of the Abstract from the original submission.

According to the suggestion, a sentence of “The recorded brain waves represent oscillatory synchrony of electrical activity generated by the varied spatial sizes of neuronal assemblies that are activated simultaneously.” was removed. As indicated in red in the revised manuscript, several words were added to imply an animal model and comply with other recommendations.

As the results, I believe that the Abstract summarizes the most important results and their significance more succinctly than before, and confirmed that the abstract does not exceed 300 words (177 words).

**2.** Line 199. The phrase "similar patterns in qualitative patterns" must be replaced by "similar qualitative patterns"

According to the suggestion, the phrase "similar patterns in qualitative patterns" was replaced by "similar qualitative patterns". (L.199)

**3.** Figure 3. In the caption, the arrows in panels A3 and A4 should be described or removed.

According to the suggestion, the phrase “indicated by arrows” was added in the figure legend. (L.545)

**4.** Lines 401-403. Please clarify what brain regions create the two oscillators.

According to the suggestion, a sentence of “The responsible brain regions of the coupling oscillators could be assigned to recurrent networks involving the NTS in the brainstem, especially a C1 neuronal group and the synaptically-connected neighboring areas [22] (Figs. S2 and S3).” was incorporated. (LL.404-406)

==

I sent the revised version of the original manuscript with “Revised Manuscript with Track Changes” in which changes were shown in red.

Thank you for your consideration. I look forward to hearing from you.

Sincerely,

Yoshinori Kawai, PhD

Adati Institute for Brain Study (AIBS),

Kawaguchi Saitama JAPAN, 333-0811

E-mail: ibs.stm.kwgt@gmail.com
